# PREDICTING TIME TO COGNITIVE IMPAIRMENT USING BRAIN AGE

**DOI:** 10.1093/geroni/igae098.2008

**Published:** 2024-12-31

**Authors:** Phoebe Imms, Siyu Chen, Haoqing Wang, Ruixi Li, Andrei Irimia

**Affiliations:** University of Southern California, Leonard Davis School of Gerontology, Los Angeles, California, United States; University of Southern California, Los Angeles, California, United States; University of Southern California, Los Angeles, California, United States; Columbia University Irving Medical Center, New York, New York, United States; University of Southern California, Los Angeles, California, United States

## Abstract

The neurological changes that occur during the asymptomatic pre-clinical phase of cognitive impairment (CI) can be quantified using brain age (BA) measures and leveraged to identify those at risk of clinical manifestation. We employ a machine learning survival model to predict future CI among healthy older adults using cognitive, demographic, genetic, and neuroimaging data including regional brain volume and anatomically specific measures of BA. Participants were 2,718 adults from NACC (1,795 females, mean age=69.14±10.91). Converters (N=343) were diagnosed with CI during the study, and non-converters (N=2,375) were CN across all visits. We predicted time to conversion (days) from baseline MRIs (185 brain volumes and regional BA measures) and 69 clinical variables. The model was trained on 1,846 converters and 252 non-converters, validated on 32 converters and 238 non-converters, and tested on 150 converters and 200 non-converters with 5-fold cross-validation. The model’s predictive accuracy (c-index) was 0.82 for training, 0.83 for validation, and 0.78 in the test set. Important features for prediction included age, neurocognitive test scores, education, history of hypertension and stroke, BA, and volumes of the right hippocampus, right precentral gyrus, bilateral white matter, and left inferior temporal gyrus. Products of this model allow us to compare a) the risk, and b) the BA biomarkers across population subgroups with heightened CI risk such as racial minorities and people with fewer years of education. The ability to accurately predict future cognitive impairment (CI) benefits those most in need of therapeutic interventions and reduces the burden of CI on healthcare systems.

